# Overexpression of a Functional *Vicia sativa* PCS1 Homolog Increases Cadmium Tolerance and Phytochelatins Synthesis in Arabidopsis

**DOI:** 10.3389/fpls.2018.00107

**Published:** 2018-02-06

**Authors:** Xingxing Zhang, Haiyun Rui, Fenqin Zhang, Zhubing Hu, Yan Xia, Zhenguo Shen

**Affiliations:** ^1^College of Life Sciences, Nanjing Agricultural University, Nanjing, China; ^2^College of Pharmacy and Chemistry and Chemical Engineering, Taizhou University, Taizhou, China; ^3^College of Agriculture and Biotechnology, Hexi University, Zhangye, China

**Keywords:** *VsPCS1*, phytochelatins, Cd, tolerance, Arabidopsis

## Abstract

Phytochelatins (PCs) catalyzed by phytochelatin synthases (PCS) are important for the detoxification of metals in plants and other living organisms. In this study, we isolated a *PCS* gene (*VsPCS1*) from *Vicia sativa* and investigated its role in regulating cadmium (Cd) tolerance. Expression of *VsPCS1* was induced in roots of *V. sativa* under Cd stress. Analysis of subcellular localization showed that VsPCS1 was localized in the cytoplasm of mesophyll protoplasts of *V. sativa*. Overexpression of *VsPCS1* (*35S::VsPCS1*, in wild-type background) in *Arabidopsis thaliana* could complement the defects of Cd tolerance of *AtPCS1*-deficent mutant (*atpcs1*). Compared with *atpcs1* mutants, *35S::VsPCS1/atpcs1* (in *AtPCS1*-deficent mutant background) transgenic plants significantly lowered Cd-fluorescence intensity in mesophyll cytoplasm, accompanied with enhanced Cd-fluorescence intensity in the vacuoles, demonstrating that the increased Cd tolerance may be attributed to the increased PC-based sequestration of Cd into the vacuole. Furthermore, overexpressing *VsPCS1* could enhance the Cd tolerance in *35S::VsPCS1*, but have no effect on Cd accumulation and distribution, showing the same level of Cd-fluorescence intensity between *35S::VsPCS1* and wild-type (WT) plants. Further analysis indicated this increased tolerance in *35S::VsPCS1* was possibly due to the increased PCs-chelated Cd in cytosol. Taken together, a functional PCS1 homolog from *V. sativa* was identified, which hold a strong catalyzed property for the synthesis of high-order PCs that retained Cd in the cytosol rather the vacuole. These findings enrich the original model of Cd detoxification mediated by PCS in higher plants.

## Introduction

Heavy metal contamination is a predominant environmental issue in the world. Cadmium (Cd) is one of highly toxic metals for all organisms and is of particular concern to human health since Cd can readily uptake by plant roots from polluted soils and transported to shoots (Wagner, 1993). This element enters the environment mainly through mining operations, smelting of metals, electroplating, municipal wastes, and phosphate fertilizers. Excess Cd can inhibit numerous biochemical and physiological processes in plants including photosynthesis and pigment synthesis, respiration, nitrogen and protein metabolism, nutrient uptake, transpiration, and plant–water relationships (Clemens, 2006).

To cope with an exposure to toxic levels of heavy metals, several mechanisms have been developed for metal detoxification, including exclusion, compartmentalization, chelating, and binding to organic ligands such as organic acids, amino acids, phytochelatins (PCs), and metallothioneins (MTs). Numerous studies have demonstrated the critical roles of PCs in metal tolerance and translocation in plants exposed to Cd, As, Hg, Pb, and Zn (Tennstedt et al., 2009; Mendoza-Cozatl et al., 2011; Franchi et al., 2014; Song et al., 2014; Shi et al., 2017; Xia et al., 2018). PC is a family of peptides with the general structure (Glu-Cys)_n_-Gly, where *n* is in the range of 2–11 (Cobbett and Goldsbrough, 2002). They form stable metal complexes and are subsequently sequestrate from the cytosol into vacuoles. PCs are synthesized directly from reduced glutathione (GSH) by the enzyme phytochelatin synthase (PCS). PCS is constitutively expressed, but is activated by metal ions, especially Cd^2+^ (Degola et al., 2014). *PCS* genes have been identified in some plants including Arabidopsis (Ha et al., 1999), *Oryza sativa* (Das et al., 2017), *Triticum aestivum* (Wang et al., 2012), *Nelumbo nucifera* (Liu et al., 2012), *Lotus japonicas* (Ramos et al., 2007), and *Ceratophyllum demersum* (Shukla et al., 2012). PC-deficient mutants of Arabidopsis and yeast are hypersensitive to Cd (Cobbett, 2000). In Arabidopsis, γ-glutamylcysteine synthetase (*γ-ECS*) and glutathione synthetase (*GS*) mutant plants were hypersensitive to Cd owing to its lower content of GSH and PC (Jobe et al., 2012). *atpcs1* was firstly identified as a knockout mutant of *PCS1* in Arabidopsis, and sensitive to Cd and As (Nahar et al., 2014). Heteroexpression of *PCS* coming from various species increased PC levels, Cd tolerance and Cd accumulation in Arabidopsis (Guo et al., 2008; Liu et al., 2012) and *Nicotiana tabacum* (Pomponi et al., 2006; Liu et al., 2011). In contrast, some studies showed that increased PCs production is not the primary tolerance mechanism to Cd (De Knecht et al., 1992; Wójcik et al., 2015), indicating a complicated mechanism underlying the PCs involved Cd tolerance.

The legume *Vicia sativa* is ordinarily farmed as food for humans and fodder for livestock. It also acts as green manure to improve soil fertility, especially in dry-land farming operations. Our previous studies have investigated the mechanisms of *V. sativa* responses to Cd stress, demonstrating that Cd toxicity most likely induced hydrogen peroxide (H_2_O_2_) and lignification in the roots of *V. sativa* by increasing apoplastic POD activity (Zhang et al., 2009, 2011; Rui et al., 2016). However, the molecular mechanisms underlying the Cd tolerance of *V. sativa* are unclear. In this study, we cloned a *V. sativa PCS* gene and determined its tissue expression patterns. Moreover, we ectopically overproduced *VsPCS1* in Arabidopsis and generated *35S::VsPCS1* (in wild-type background) and *35S::VsPCS1/atpcs1* (in *AtPCS1*-deficent mutant background) and examined their growth under Cd stress. The metal accumulation and distribution were also investigated in these transgenic plants. Our study aimed to unravel the physiological roles of *VsPCS1* and the Cd-tolerance mechanisms in higher plants.

## Materials and Methods

### Plant Materials, Growth Conditions, and Treatments

The ZM variety of *V. sativa* was used in this study, the culture process of plants was described as previously (Rui et al., 2016). For *VsPCS1* expression analysis, 7-day-old plants were treated with 0, 5, and 50 μM Cd for different days.

Sterile seeds of Arabidopsis wild-type (Columbia, WT), mutant *atpcs1*, transgenic plants were surface sterilized and germinated on 1/2 Murashige & Skoog (MS) agar medium (pH 5.8) containing the different concentrations of Cd. After 3 days at 4°C in the dark, seeds were germinated in a growth chamber (22/18°C day/night temperatures, 16/8 h day/night photoperiod) for 8 days, the length of plants roots, fresh weight, and dry weight were measured.

To analysis GHS and PCs content and Cd transport, the seeds of WT, *atpcs1, 35S::VsPCS1*, and *35S::VsPCS1/atpcs1* were first grown on half-strength MS agar plates for 2 weeks, then the seedlings were grown hydroponically in 1/4 strength Hoagland’s nutrient solution (Liu et al., 2017) for 2 weeks, after, the plants was placed in the solution with or without 10 μM CdCl_2_ (Remans et al., 2008; Cuypers et al., 2011) for 24 h to detect the expression level of *AtABCC1, 3* [C-type ATP-binding cassette (ABC) transporter], *AtCAX2* (Calcium exchanger 2), *AtNRAMP3* (Natural resistance-associated macrophage proteins 3), and for 5 days to analyze NPT content and intracellular Cd localization.

### Isolation of Full-Length *VsPCS1* cDNA

The nucleic acid sequences of other plant phytochelatin synthases were searched from NCBI database^1^. The sequences were analyzed using DNAMAN software (Version 6.0.3.99 LynnonBioSoft, Foster City, CA, United States). Primers were designed according to the highly conserved region of PCS1 for *VsPCS1* isolation. Total RNA was isolated by total RNA extraction Kit (TakaRa) from *V. sativa*. The first strand cDNA was synthesized by RT-PCR using PrimeScript (TaKaRa) and oligo (dT) primers, PCR amplifications were performed with *VsPCS1* primers. The PCR products were sequenced, then the sequences of *VsPCS1* were tested by BLAST^2^.

### Plants Transformation and Selection

Wild-type Arabidopsis (Col-0) and its mutant, *atpcs1* (SAIL_650_C12), with T-DNA insertion in an exon of *AtPCS1* gene was available from public repositories^3^ for this study. Homozygous *atpcs1* lines were identified by the method of Yi and An (2013).

*pCAMBIA1304* was used as the plant expression vectors. CDS of *VsPCS1* was cloned into *pCAMBIA1304* using primers of PCS1-F2 and PCS1-R2. The confirmed plasmid were transformed into Arabidopsis WT Col-0 and the mutant *atpcs1* plants via standard floral dip transformation using *Agrobacterium tumefaciens* (Clough and Bent, 1998), which were named *35S::VsPCS1* and *35S::VsPCS1/atpcs1*, respectively. Homozygotic transgenic lines of T3 progeny were used for subsequent study.

### Quantitative RT-PCR Analysis

RNA isolation and cDNA synthesis followed the procedure above mentioned. The quantitative RT-PCR was performed with SYBR pre-mix EX Taq (TaKaRa) by a Real-time PCR system (Eppendorf, Mastercycler ep realplex, Germany) with *VsPCS1* primers presented in Supplementary Table 2. *V. sativa* gene *Actin11* was used as an internal control and relative expression levels of genes which were calculated by 2^-^∆∆^C_T_^ method (Gao et al., 2016). The primers for *AtABCC1, 3, AtCAX2, AtNRAMP3* genes presented in Supplementary Table 1, and *AtActin2* gene was used as an internal control.

### Protoplast Preparation

The mesophyll protoplasts of WT, *atpcs1, 35S::VsPCS1*, and *35S::VsPCS1/atpcs1* were isolated by enzyme solution containing 0.25% w/v macerozyme R-10 (Yakult), 1% w/v cellulase R-10 (Yakult), 0.4 M D-mannitol, 20 mM MES (pH 5.7), 10 mM CaCl_2_, 20 mM KCl, and 0.1% w/v bovine serum albumin (Yoo et al., 2007). The isolated cells were purified using 21% sucrose and counted on a hemacytometer.

*V. sativa* protoplasts were prepared from 14-days-old leaves using the same modifications as described (Wu et al., 2009). The isolated cells were purified and concentrated using 30% sucrose.

### Subcellular Localization of VsPCS1

35S (CaMV35S) promoter-driven expression clones were generated in the *pSGFP* vectors, resulting in N-terminal green fluorescent protein (GFP)-protein fusions. The CDS of *V. sativa PCS1* gene amplified using the primers of PCS1F1 and PCS1R1. PCR products were cloned into *pSGFP* vector and confirmed by sequencing. *35S::VsPCS1-GFP* plasmid were transiently expressed in *V. sativa* mesophyll protoplasts via polyethylene glycol (4000)-calcium transfection (Yoo et al., 2007). Incubating at room temperature for 16 h, transformed cells were observed with a uitraviewvox confocal microscope (PerkinElmer, United States).

### Determination of Cd Content

The plant materials were washed with 5 mM CaCl_2_ for half an hour, and then dried at 105°C for 15 min and 48 h at 80°C in an oven. The digestion of dried samples according to the method of Rui et al. (2016). Cd concentrations were determined using atomic absorption spectrophotometer (novAA^®^ 400; Analytik Jena, Jena, Germany).

### GSH and PCs Analysis

For Cys, GSH and PCs measurement, they were extracted by 1 ml TFA (0.1%) and 6.3 mM diethylene triaminepentaacetic acid, put the homogenate on ice for 15 min. Following centrifugation (13200 *g*, 30 min, 4°C), the supernatant were derivatized based on the methods of Kühnlenz et al. (2015). UPLC system (Agilent 1290 Infinity, Germany) was equipped with a ZORBAX Eclipse Plus C18 column (2.1 mm × 100 mm) used for the separation of the mBBr-labeled thiols. Thiols were detected using the fluorescence detector set at excitation and emission wavelengths of 380 and 470 nm. Quantification was performed via authentic Cys, GSH, PC_2_, PC_3_, and PC_4_ standards.

### Intracellular Cd Localization through Cd-Sensing Fluorescent Dyes

The leaf protoplasts with or without Cd treatment from WT, *atpcs1, 35S::VsPCS1*, and *35S::VsPCS1/atpcs1* were loaded with 0.04% v/v Leadmium Green AM dye (Molecular Probes, Invitrogen, Carlsbad, CA, United States). Intracellular Cd localization was observed according to the method of Park et al. (2012) using a uitraviewvox confocal microscope (PerkinElmer, United States). The intensity of green fluorescence signal was quantified by ImageJ software.

### Statistical Analysis

The data were analyzed by one-way analysis of variance, followed by multiple comparisons with the least significant difference (LSD) test (*P* < 0.05), using SPSS software (ver. 17.0; SPSS, Inc., Chicago, IL, United States), different letters indicate significant differences among treatments.

## Results

### Isolation of Full-Length *VsPCS1* Gene

**FIGURE 1:**
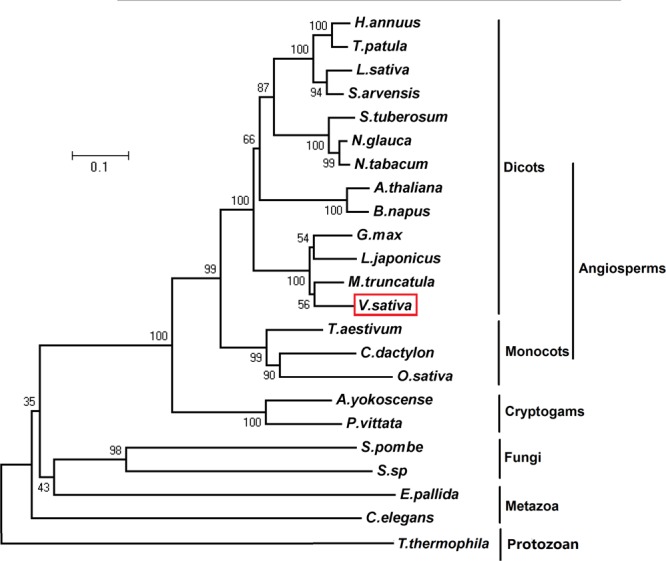
Phylogenetic analysis of PCS1. Phylogenetic relationship of PCS1 from *Vicia sativa, Athyrium yokoscense* (BAB64932.1), *Arabidopsis thaliana* (AAD50593.1), *Brassica napus* (AOV94290.1), *Cynodon dactylon* (AAO13810.2), *Caenorhabditis elegans* (NP_001122615.1), *Exaiptasia pallida* (KXJ16980.1), *Glycine max* (NP_001235576.1), *Helianthus annuus* (OTG28437.1), *Lactuca sativa* (AAU93349.1), *Lotus japonicus* (AAQ01752.1), *Medicago truncatula* (XP_013449920.1), *Nicotiana glauca* (ABX10958.1), *Nicotiana tabacum* (AAO74500.1), *Oryza sativa* (AAO13349.2), *Pteris vittata* (ADR51707.1), *Sonchus arvensis* (ACU44656.1), *Schizosaccharomyces pombe* (NP_593552.1), *Solanum tuberosum* (NP_001275308.1), *Sporobolomyces* sp. (AIS24729.1), *Tagetes patula* (AQT18915.1), *Tetrahymena thermophila* (AAY68362.2), and *Triticum aestivum* (AAD50592.1). The phylogenetic tree was constructed by neighbour-joining (NJ) method of MEGA5.01 with 1000 bootstraps replicates.

The complete full-length phytochelatin synthase cDNA (complementary DNA) was amplified from *V. sativa* cDNA using a pair of degenerate primers. *VsPCS1* coding DNA sequence (CDS) contains 1500 base pairs that encodes 500 amino acids (Supplementary Data 1, 2). Sequences analysis of amino acids showed that PCS1 is highly conserved between *V. sativa* (VsPCS1) and *Medicago truncatula* (MtPCS1) with 89.4% identity and 94% similarity. Twenty Cys residues are present in VsPCS1. Seven Cys residues are conserved in plant kingdom, and one Cys specific residue is in VsPCS1 (Supplementary Figure 1). In the aspect of catalytic activity, VsPCS1 holds the same catalytic active sites as other plant PCS1. Phylogenetic analysis for PCS1 demonstrated that *V. sativa* was grouped with leguminous plants. These data suggested that the VsPCS1 obtained is a *V. sativa* PCS1 homolog (**Figure 1**).

### Expression Patterns of *VsPCS1* in *V. sativa*

**FIGURE 2:**
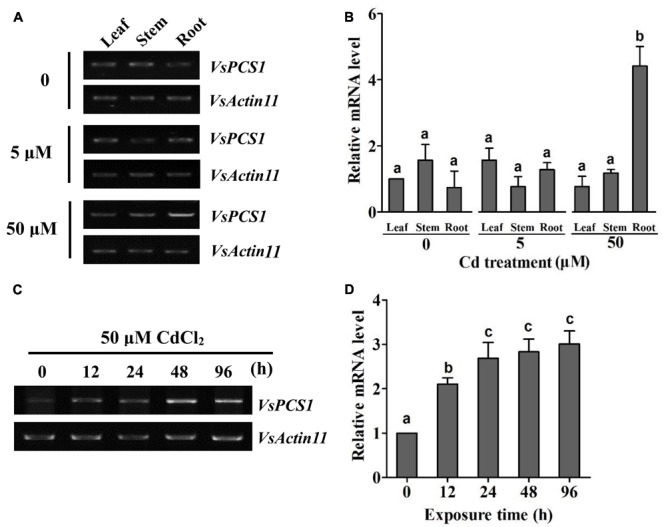
Expression analysis of *VsPCS1* in *V. sativa*. RT-PCR **(A)** and quantitative RT-PCR **(B)** analysis the expression level of *VsPCS1* in different organs (roots, stems, and leaves) of *V. sativa* under Cd treatment. RT-PCR **(C)** and quantitative RT-PCR **(D)** analysis the expression level of *VsPCS1* under 50 μM Cd treatment for different time in roots of *V. sativa*. *VsActin11* was used as an internal control. Values are means ± SD of three biological replicates. Columns labeled with distinct lowercase letters indicate statistically significant differences among treatments (*P* ≤ 0.05).

PCS homologs have been characterized as crucial factors for the detoxification of metals in organisms (Pomponi et al., 2006; Liu et al., 2011; Shukla et al., 2012; Kühnlenz et al., 2014, 2015). To test whether *VsPCS1* hold the function to detoxify the heavy metals in *V. sativa*, we firstly determined the expression of *VsPCS1* in response to excess Cd among different tissues, including leaves, stems, and roots. Through employing reverse transcript-polymerase chain reaction (RT-PCR) and quantitative RT-PCR, no significant difference on the *VsPCS1* transcripts was detected in all the detected tissues under Cd-free and 5 μM Cd treatments. However, 50 μM Cd treatment dramatically induced the increase of *VsPCS1* transcripts in roots, being about 4.2 times of Cd-free treatment (**Figures 2A,B**). Such Cd induced increase was not observed in leaves and stems under 50 μM Cd treatment for 24 h. Time-course analysis demonstrated that *VsPCS1* in the roots rapidly and consistently responded to 50 μM Cd, showing a significant increase of transcript from 12 to 96 h (**Figures 2C,D**).

### Subcellular Localization of VsPCS1 in *V. sativa* Mesophyll Protoplasts

**FIGURE 3:**
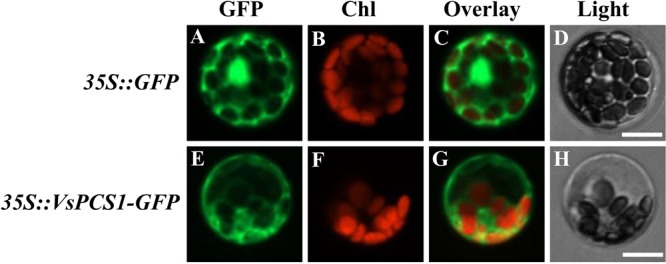
Subcellular localization of VsPCS1. *V. sativa* protoplasts transformed with *35S::GFP* **(A–D)** or *35S::VsPCS1-GFP* **(E–H)** plasmids. Confocal cross sections show green fluorescent protein (GFP) fluorescence **(A,E)**, chlorophyll auto-fluorescence **(B,F)** and the overlay of GFP and chlorophyll auto-fluorescence **(C,G)**. In **(D,H)** depicts the corresponding bright-field images. Bars = 14 μm.

To investigate the subcellular localization of VsPCS1, a fused protein of VsPCS1 with GFP was transiently expressed in mesophyll protoplasts of *V. sativa*. GFP signal mainly accumulated in the cytoplasm of VsPCS1-GFP transformed cells, whereas GFP signal is diffused everywhere in GFP transformed cells (**Figure 3**).

### Ectopic Expression of *VsPCS1* Could Rescue the Cd Tolerance of Arabidopsis *PCS1* Deficient Mutant

**FIGURE 4:**
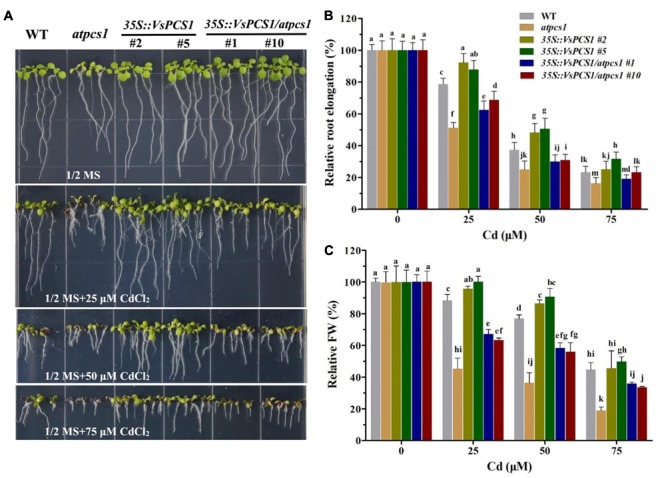
The *VsPCS*1 transgenic Arabidopsis shows tolerance to Cd toxicity. Seedlings grown in half-strength MS medium treated with or without of CdCl_2_ for 8 days **(A)**. Relative root length **(B)** and fresh weight **(C)** of each genotype on plate treated with or without CdCl_2_. Values are means ± SD (*n* = 30–50). Columns labeled with distinct lowercase letters indicate statistically significant differences among treatments (*P* ≤ 0.05).

To test whether VsPCS1 is a functional homolog of Arabidopsis PCS1, we conducted a complementation experiment by ectopic expression of *VsPCS1* in Arabidopsis *PCS1* deficient mutant *atpcs1* (designated as *35S::VsPCS1/atpcs1*). Consistent with previous report (Nahar et al., 2014), *atpcs1* was hypersensitive to Cd. Such Cd hypersensitivity of *atpcs1* was restored when constitutively expressed *VsPCS1* (**Figure 4**).

### Cd Tolerance in *VsPCS1*-Overexpressing Arabidopsis

To further determine the function of *VsPCS1* in detoxifying Cd, *VsPCS1* was also ectopically expressed in wild-type Arabidopsis (Col-0). Fifteen independent *35-VsPCS1* lines were obtained by using hygromycin screen; six out of seven independent transgenic lines showed *VsPCS1* overexpression; and two transgenic lines (#2 and #5) were selected for further analysis (Supplementary Figure 2). Ectopic expression of *VsPCS1* has no effect on the levels of *AtPCS1* in Arabidopsis (Supplementary Figure 3). To investigate Cd tolerance of these *VsPCS1*-expressing lines, Arabidopsis seeds of WT and homozygotic transgenic lines were germinated and grown on solid 1/2 MS medium contained 0, 25, 50, and 75 μM Cd for 8 days. No significant difference could be observed in the growth between WT and all of transgenic lines under Cd-free treatment (**Figure 4A**). When treated with 25 and 50 μM Cd, *35S::VsPCS1* (*#2* and *#5*) had a longer root length and more fresh weight than WT (**Figures 4B,C**). These results demonstrate that overexpression of *VsPCS1* increase Cd tolerance in *35S::VsPCS1*.

### Cd Accumulation in *VsPCS1*-Overexpressing Arabidopsis

**FIGURE 5:**
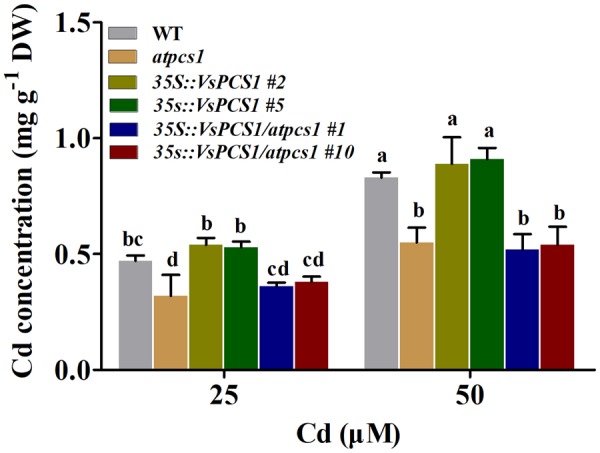
Effects of overexpression *VsPCS*1 on cadmium accumulation in Arabidopsis. Seedlings grown in half-strength MS medium in the presence or absence of CdCl_2_ for 8 days. Values are means ± SD of three biological replicates, columns labeled with distinct lowercase letters indicate statistically significant differences among treatments (*P* ≤ 0.05).

Cd tolerance is tightly related to the Cd uptake from growth medium (Lin and Aarts, 2012). Since Cd tolerance of both *35S::VsPCS1* and *35S::VsPCS1/atpcs1* significantly increased, we investigated the effects of *VsPCS1* overexpression on Cd concentration. Under control condition, Cd concentration was undetectable in all Arabidopsis lines (data not shown). After 8-day treatment with 25 and 50 μM Cd, a comparable Cd concentration was detected in WT and *35S::VsPCS1*. Similarly, no difference in Cd concentration was observed between *atpcs1* and *35S::VsPCS1/atpcs1* (**Figure 5**).

### Cellular Distribution of Cd in *VsPCS1*-Overexpressing Arabidopsis

**FIGURE 6:**
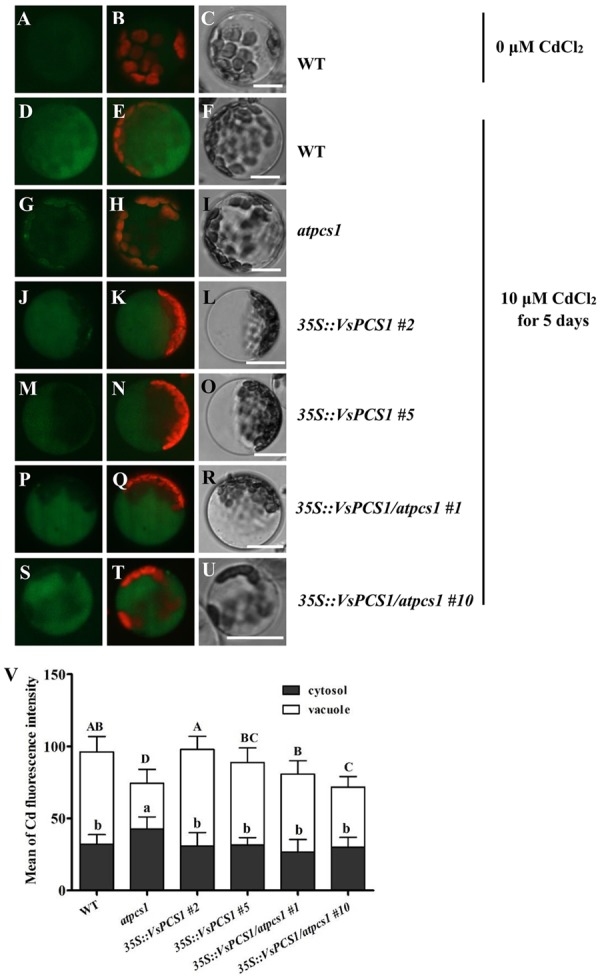
Analyses of cytosolic and vacuolar Cd in Arabidopsis leaf protoplasts. **(A–C)** WT protoplast treated without Cd. WT **(D–F)**, *atpcs1* **(G–I)**, *35S::VsPCS1* **(J–O)** and *35S::VsPCS1/atpcs1* **(P–U)**, protoplasts isolated from plants grown in10 μM CdCl_2_ for 5 days. Leadmium^TM^ Green signal was detected in protoplasts of the WT **(D**), *35S::VsPCS1* **(J,M)**, *35S::VsPCS1/atpcs1* **(P,S)**, and *atpcs1* mutant **(G)**. Merged images of green Leadmium^TM^ Green and red chlorophyll auto-fluorescence **(B,E,H,K,N,Q,T)** and bright-field images **(C,F,I,L,O,R,U)** are shown. Scale bars = 14 μm. **(V)** Fluorescence signal intensity in the cytosol and in the vacuole, values are means ± SD (*n* = 15–20), and columns labeled with distinct lowercase letters or uppercase indicate statistically significant differences in cytosol or in vacuole among treatments, respectively.

Apart from Cd uptake, the tolerance is also affected by cellular distribution of Cd. To examine whether the enhanced Cd tolerance conferred by overexpression of *VsPCS1* resulted from the increased vacuolar sequestration, the cellular distribution of Cd was analyzed in the leaves of WT, *atpcs1, 35S::VsPCS1* (*#2* and *#5*), and *35S::VsPCS1/atpcs1* (*#1* and *#10*) using a Cd-indicator dye, by which Cd concentration could be indicated by green fluorescence. In the absence of Cd, leaf cells from WT showed a negligible Leadmium^TM^ Green fluorescence signal (**Figures 6A–C**). After Cd treatment, observed green fluorescence was emitted in all of the detected lines. As expected, the green fluorescence signal mainly emitted from the vacuole of cells in wild-type (**Figures 6D–F**) and transgenic lines (*35S::VsPCS1 #2, 35S::VsPCS1#5, 35S::VsPCS1/atpcs1#1*, and *35S::VsPCS1/atpcs1#10*) (**Figures 6J–U**). However, the green fluorescence signal in *atpcs1* was detected mainly in cytoplasm and chloroplasts, being an orange-green signal when green Leadmium^TM^ Green and red chlorophyll auto-fluorescence were merged (**Figures 6G–I**). Quantitative analysis showed that average intensity of Cd-fluorescence was significantly lower in the vacuole and higher in the cytosol of *atpcs1* than *35S::VsPCS1/atpcs1*, demonstrating that *VsPCS1* could rescue the defects of PC-based sequestration of Cd into the vacuole in *atpcs1*. Surprisingly, no difference in average intensity of Cd-fluorescence was observed between WT and *35S::VsPCS1* (**Figure 6V**).

**FIGURE 7:**
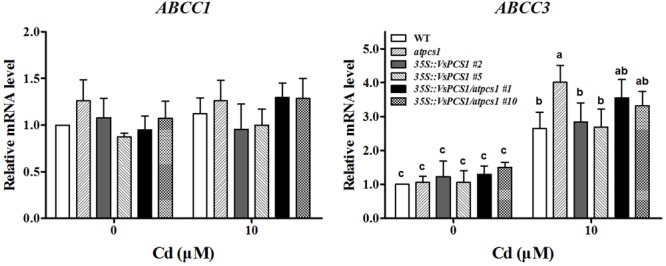
Effects of Cd treatment on gene expression of *AtABCC1* and *AtABCC3* in different Arabidopsis. Seedlings were exposed to 0 and 10 μM CdCl_2_ for 24 h. *AtActin2* was used as an internal control. Values are means ± SD of three biological replicates. Columns labeled with distinct lowercase letters indicate statistically significant differences among treatments (*P* ≤ 0.05).

The PC-Cd complexes sequestrating to vacuole were mainly mediated by two ABCC proteins, *AtABCC1* and *AtABCC3* in Arabidopsis (Park et al., 2012; Brunetti et al., 2015). To exclude the possibility that the rescued Cd vacuole transport by overproducing *VsPCS1* is owing to the increased ABCC proteins in Arabidopsis, we measured their expression in different Arabidopsis lines. As shown in **Figure 7**, the expression of *AtABCC1* and *AtABCC3* had the same transcription level in all lines under control condition. Although the transcript level of *AtABCC3* increased in all lines after 10 μM Cd treatment, no significant increase were observed between VsPCS1 overproducing lines and WT. Unexpectedly, a higher *AtABCC3* transcript was detected in the *atpcs1* than other lines. Cd treatment did not affect the expression of *AtABCC1*.

### Content of PC in *VsPCS1*-Overexpressing Arabidopsis

**FIGURE 8:**
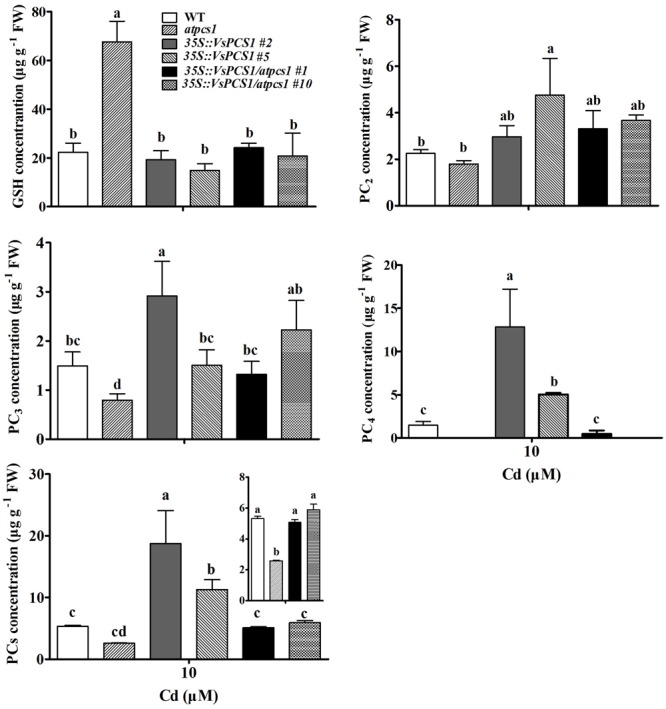
The content of Cys, GSH, PC_2_, PC_3_, PC4, and total PCs under Cd treatment in Arabidopsis. Seedlings were treated with 10 μM CdCl_2_ for 5 days. Values are means ± SD of three biological replicates, and columns labeled with distinct lowercase letters indicate statistically significant differences among treatments (*P* ≤ 0.05).

Phytochelatin synthases are well-known to synthesize PCs from reduced glutathione (Noctor et al., 2011). To test whether PCS1 hold the catalytic activity for PCs synthesis, we measured the GSH and its high-order products PCs in the lines we used. As expected, *atpcs1* mutant had the highest contents of GSH among all the detected lines. Consistently, a relative reduction of PCs was observed in *atpcs1* compared with WT. Such change could be bounced back by introducing *VsPCS1* into *atpcs1* (**Figure 8**). Total PCs content in *35S::VsPCS1/atpcs1* was about twofold of that in the mutant *atpcs1*. Surprisingly, overexpressing *VsPCS1* in Arabidopsis dramatically increase high-order products, especially PC_4_ in *35S::VsPCS1*.

## Discussion

Phytochelatin synthases encoded by *PCS* genes. It have been identified that PCS has a role in the detoxification of diverse metals and metalloids (Grill et al., 1985; Ha et al., 1999). In this study, a phytochelatin synthase cDNA (*VsPCS1*) was isolated from *V. sativa* and the alignment of the amino acid sequences of VsPCS1 and other plant species was analyzed. As shown in Supplementary Figure 1, N-terminal amino acid residues of the plant PCS1 shows high conservation for approximately the first 221 amino acid residues, whereas the C-terminal, which contains rich cysteine residues, exhibits large variability, indicating that N-terminal is essential part for PCS1 function in plants. In support, three key amino acids, Cys56, His162, and Asp180 were located at the N-terminal of VsPCS1, the corresponding amino acids of which in AtPCS1 have been identified to be essential for AtPCS1 catalytic activity and Cd tolerance (Cobbett and Goldsbrough, 2002; Vivares et al., 2005). Additionally, compared with PCS1 in other plant species, a Cys16 residue only in VsPCS1 possibly hold a specific role in *V. sativa*.

It has been reported that *AtPCS1* and *AtPCS2* were observed to be constitutively expressed rather transcriptionally regulated by Cd (Ha et al., 1999; Cazalé and Clemens, 2001). In contrast, some studies reported that the transcriptional expression of *PCS* is regulated when exposed to heavy metals or metalloids (Ramos et al., 2007; Das et al., 2017). In this study, the *VsPCS1* are expressed in leaves, stems and roots of *V. sativa*. When *V. sativa* was treated with 50 μM Cd, a significant induction of *VsPCS1* expression was observed in root but not in stems and leaves (**Figure 2**). Similar expression pattern was reported for a rice *PCS1* homolog, *OsPCS1* under Cd stress (Das et al., 2017). This Cd-induced expression of *PCS1* only in root may be the fact that roots are the major site of metal(loid)s accumulation in non-hyperaccumulator plants (Hernández et al., 2015).

Cytoplasm is a major formation site for the low molecular weight complexes of Cd and PC that are in turn transported into the vacuole to generating high molecular weight Cd and PC complexes (Cobbett, 2000). Consistently, VsPCS1 proteins were located in the cytoplasm. Similar cellular localization of AtPCS1 was observed in Arabidopsis (Blum et al., 2010). Besides cytoplasm localization of PCS1, other kind of cellular localization was also reported for PCS1 homolog. For an instance, a recent study indicates that SpPCS1 protein from *Schizosaccharomyces pombe* was localized to the mitochondria (Shine et al., 2015). This different cellular localization of PCS1 homolog among different species suggested that PCS1 might have the diversified roles in metal transport.

A decreased tolerance has been reported in mutant *atpcs1* when exposed on Cd and As (Nahar et al., 2014). Similarly, a compromised Cd tolerance in *atpcs1* mutant was observed in this study when compared with WT (**Figure 4**). Overexpressing *VsPCS1* in the *AtPCS1*-deficent mutant *atpcs1* can increase Cd tolerance. A similar increase of Cd tolerance in *35S::VsPCS1* (**Figure 4**). This increased Cd tolerance might be due to the elevated content of PCs under Cd stress because PCs contents is positively related Cd tolerances among different lines we tested (**Figure 8**). Furthermore, *VsPCS1* homologs have been identified to catalyze the PCs synthesis and execute important role for the detoxification of metals in plants and other living organisms (Kühnlenz et al., 2015; Zanella et al., 2016). This increased Arabidopsis Cd tolerance was also reported by heteroexpression of PCS1 from *Allium sativum* (Guo et al., 2008) and *Nelumbo nucifera* (Liu et al., 2012). Additionally, tobacco overexpressing *AtPCS1* and *TcPCS1* from *Thlaspi caerulescens* also displayed increased Cd tolerance (Pomponi et al., 2006; Liu et al., 2011). By contrast, overproducing *AtPCS1* in Arabidopsis reduced Cd tolerance despite enhanced PC production (Lee et al., 2003). This contradictory results on Cd tolerance among various transgenic plants might be due to the sequence variation of PCS genes from various sources or the difference in experimental conditions.

Apart from PCs contents, the increased Cd tolerance might be related to the availability of GSH. Pomponi et al. (2006) reported a direct correlation between the availability of GSH and the increase in Cd tolerance and accumulation in AtPCS1 overexpressing tobacco plants. Exogenous GSH increased the Cd tolerance of overexpressing *AtPCS* tobacco, but did not increase the Cd tolerance of overexpressing *AtPCS* Arabidopsis (Brunetti et al., 2011). Brunetti et al. (2011) observed that the endogenous content of PCs and GSH could affect Cd tolerance in *AtPCS1* overexpressing Arabidopsis and tobacco. GSH functions as an antioxidant by scavenging free radicals and protects cells from the oxidative stress induced by heavy metals. GSH also serves as a direct precursor for PC synthesis. Some studies showed that Cd stress resulted in depletion of GSH, subsequently caused oxidative damage (Semane et al., 2007; Bankaji et al., 2015). In our study, the increase in PC synthesis did not cause a depletion of GSH in *VsPCS1-*expresing lines. No difference in GSH concentration was observed among all lines exposed to Cd except for *atpcs1* (**Figure 8**), in which GSH was significantly accumulated due to inhibition of PC synthesis. The unchanged level of GSH, combined with the increased PC, showed that these transgenic plants had ability to restore the GSH pool used for PC synthesis.

Compared with that in *atpcs1*, average intensity of Cd-fluorescence in mesophyll cytoplasm was significantly lower in *35S::VsPCS1/atpcs1* lines, accompanied with higher intensity of Cd-fluorescence in vacuole, indicating that a role of *VsPCS1* is involved in PC-based sequestration of Cd into the vacuole. However, this increased Cd sequestration into the vacuole was not observed in *35S::VsPCS1* lines although higher PCs contents were detected. Such difference might be explained by a higher PC_4_ in *35S::VsPCS1* than *35S::VsPCS1/atpcs1* under Cd stress. PCs with higher degree of polymerization were more efficient in the complexation of Cd (Gupta and Goldsbrough, 1991) and the small chain length peptide-Cd complexes can be more easily transported across the tonoplast than the longer peptide-Cd complexes (Shukla et al., 2013). *VsPCS1* induced production of PC_4_ formed longer peptide-Cd complexes that could not transported across the tonoplast, finally resulting in no increased Cd sequestration into the vacuole in *35S::VsPCS1*. Additionally, a high PC_4_ content and a proportion in the total PCs in *35S::VsPCS1* indicates that *VsPCS1* hold a strong catalyzed property for the synthesis of high-order PCs, especially PC_4_. Surprisingly, no or less PC_4_ was detected in *35S::VsPCS1/atpcs1*. Possibly, the loss of *AtPCS1* in *35S::VsPCS1/atpcs1* lead to the deficiency of the precursors for high-order PCs, PC_4_. Up to date, only AtPCS1 was reported to promote the synthesis of PC_4_ under Cd stress (Brunetti et al., 2011).

Another possible explanation is that the subsequent sequestration of PC-Cd complexes from the cytosol into vacuoles might be limited by other factors such as the activity of vacuolar transporters. It has been reported that tonoplast transporters play a crucial role in transport of PCs-Cd complexes (Adamis et al., 2007; Park et al., 2012; Brunetti et al., 2015). Guo et al. (2012) reported that simultaneous expression of AsPCS1 and YCF1 (yeast cadmium factor 1, a member of vacuolar ATP-binding cassette transporter family) in Arabidopsis increased the tolerance and accumulation of Cd and As. Cd can be pumped directly into vacuoles by members of CAX (cation exchange transporters) family and HMA3 (Heavy metal ATPase 3) (Gravot et al., 2004; Mendoza-Cozatl et al., 2011). Cd can also be released from the vacuole by NRAMP-type transporters, *AtNRAMP3* and *AtNRAMP4* (Oomen et al., 2009). In this study, the transcription levels of *AtCAX2* and *AtNRAMP3* was not affected by Cd treatment in all lines (Supplementary Figure 4), which is in agreement with observations of Hirschi et al. (2000) and Oomen et al. (2009). Furthermore, although a lower Cd distribution is in vacuoles of *atpcs1* than those of WT and *35S::VsPCS1/atpcs1* lines, *atpcs1* mutant had higher transcript of *ABCC3* than WT and a comparable level of *35S::VsPCS1/atpcs1* lines, suggesting the expression difference in ABCC transporters might not explain different patterns of Cd distribution in *35S::VsPCS1* and *35S::VsPCS1/atpcs1* lines.

## Conclusion

We isolated a functional PCS1 homolog from *V. sativa* that located in the cytoplasm. Ectopic expressing of *VsPCS1* in Arabidopsis increase Cd tolerance in *35S::VsPCS1* and *35S::VsPCS1/atpcs1*, which is positively correlated with PC contents in plants. Surprisingly, *VsPCS1* exhibited a strong catalyzed property for the synthesis of high-order PCs. Such property might be explained that a promoting effects on Cd transport into vacuole by overexpressing *VsPCS1* in the *35S::VsPCS1/atpcs1*, but not in the *35S::VsPCS1*.

## Author Contributions

XZ, ZS, and YX conceived, designed the experiments. ZS, XZ, YX, and ZH analyzed the data and revised the manuscript. FZ provided plants materials. XZ and HR carried out the experiments. All the authors read and approved the manuscript.

## Conflict of Interest Statement

The authors declare that the research was conducted in the absence of any commercial or financial relationships that could be construed as a potential conflict of interest.

## Footnotes

1 http://www.ncbi.nlm.nih.gov/

2 http://blast.ncbi.nlm.nih.gov/Blast.cgi

3 http://www.Arabidopsis.org/
