# Supplementary material for: Overexpression of a Functional Vicia sativa PCS1 Homolog Increases Cadmium Tolerance and Phytochelatins Synthesis in Arabidopsis
Source: Front Plant Sci. 2018 Feb 6;9:107. doi: 10.3389/fpls.2018.00107 (PMC5808204; doi:10.3389/fpls.2018.00107)
Supplement: Supplementary file 1 [file Data_Sheet_1.DOCX]

Supplementary Material

Overexpression of a *Vicia sativa* PCS1 homologue confers Cd tolerance in Arabidopsis wild type and *AtPCS1*-deficent mutant with different mechanism

**Xingxing Zhang^1^, Haiyun Rui^2^, Fenqin Zhang^3^, Zhubing Hu^1^, Zhenguo Shen ^1^, Yan Xia ^1*^**

*** Correspondence:** **Yan Xia**: yxia@njau.edu.cn

# Supplementary Data

**Supplementary Data 1** Sequences of *VsPCS1* cDNA

ATGGCGATGGCGGGGTTGTATAGGCGCCTTCTTCCTTCTCCCCCTTGCTTTGATTTTGCGTCATCTCATGGCAAGCAACTTTTTGTTGAAGGCATTCAAAATGGAACTATGGAAGGCTTTTATAGATTGATATCTTATTTCCAAACACAATCCGAACCCGCCTATTGTGGTCTCGCCAGTCTCGCCATGGTGCTCAATGCTCTTGCCATTGATCCGGGCAGGAAATGGAAAGGACCTTGGAGATGGTTCGATGAATCCATGTTGGATTGTTGCGAACCTTTAGAAATCGTGAAAGCTAGAGGTATCACATTTGGTAAACTCGTATGCTTGGCTCATTGTGCTGGAGCCAAAGTGGACGCCTTTCATGCTAGTCGGAGCAACATCAATGAATTTCGTAAATATGTCCTCAAGTGTTCAACCTCTGATAACTGTCATTTAATCTCATCCTACCACAGAGGTGCTCTCAAACAGACGGGAACCGGTCACTTTTCTCCTATTGGAGGCTATCATGTTGGAAAGGACATGGCATTAATTTTAGATGTTGCCCGTTTTAAGTATCCTCCACATTGGGTTCCACTTACCATGCTTTGGGAAGGCATGAATGATATTGATGAATCTACCGGAAAATCTAGAGGGTTCATGCTTATATCAAGGCTGCACACGGAACCTGGCATGCTTTACACTCTGAGTTGCAAACATGAGAGTTGGAATAGTATTGCAAAGTTCCTAATGGATGATGTGCCTCTTCTATTAAAATCAGAGGATGTGGAAGACATTTACAAGGTTCTTTCAATTATATTCACATCGCTGCCATCCAATTTTGAAGAATTCATCAAATGGGTCGCAGAGGTCAGAAGGCATGAGGATGGCGATTCAAGCTTAAGTGAAGAGGAGAAAACAAGGCTCGCTGTCAAGGAAGAAGTATTGCGACACGTGCATGAAACGAGGCTTTTCAAACACGTGTCCTCTTTTCTGTCAGGTTCTTGTGGCAGACAGACAATAACTTCAGGTGACGGAAACACCTTACCTGTCATTGCTGCAAGTGTTTGTTGCCAAGGAGCAGAGATTTTAGGCGGAAAACTTAGTTCACCGGCCGCGTATTGCTGTCCAGGAACATGCGTGAAATGTTGGAAAGCCGAAGATGACAAATCAATAACAATGGTTAGTGGGACTGTGGTAAACGGTAATTCAGAGCAAGGGGTTGATGTTCTGATCCCTTCATCATCTGGGAAATTATGTTGCACTTGTTCTAGCAAAAAGAACATTAGGATGCACCCAGCTAGCACTGATGTGTTAACTGTGCTTTTACTGTCCTTGCCATCTACAACATGGGGTGGTATCACGGATGAGAAGCTTTTGAAAGAAATACATGATCTTGTTTCACATGAAAATCTTTCTACTTTACTTCAAGAAGAGGTTCTACACTTAAGACGTCAACTACATATTCTAAAGAGATGTCAAGAGGGTAAGGTAGACGAAGATCTTGATGCTCCTTCATCTTAG

**Supplementary Data 2** Sequences of deduced amino acids of VsPCS1

MAMAGLYRRLLPSPPCFDFASSHGKQLFVEGIQNGTMEGFYRLISYFQTQSEPAYCGLASLAMVLNALAIDPGRKWKGPWRWFDESMLDCCEPLEIVKARGITFGKLVCLAHCAGAKVDAFHASRSNINEFRKYVLKCSTSDNCHLISSYHRGALKQTGTGHFSPIGGYHVGKDMALILDVARFKYPPHWVPLTMLWEGMNDIDESTGKSRGFMLISRLHTEPGMLYTLSCKHESWNSIAKFLMDDVPLLLKSEDVEDIYKVLSIIFTSLPSNFEEFIKWVAEVRRHEDGDSSLSEEEKTRLAVKEEVLRHVHETRLFKHVSSFLSGSCGRQTITSGDGNTLPVIAASVCCQGAEILGGKLSSPAAYCCPGTCVKCWKAEDDKSITMVSGTVVNGNSEQGVDVLIPSSSGKLCCTCSSKKNIRMHPASTDVLTVLLLSLPSTTWGGITDEKLLKEIHDLVSHENLSTLLQEEVLHLRRQLHILKRCQEGKVDEDLDAPSS

# Supplementary Figures and Tables

## Supplementary Figures


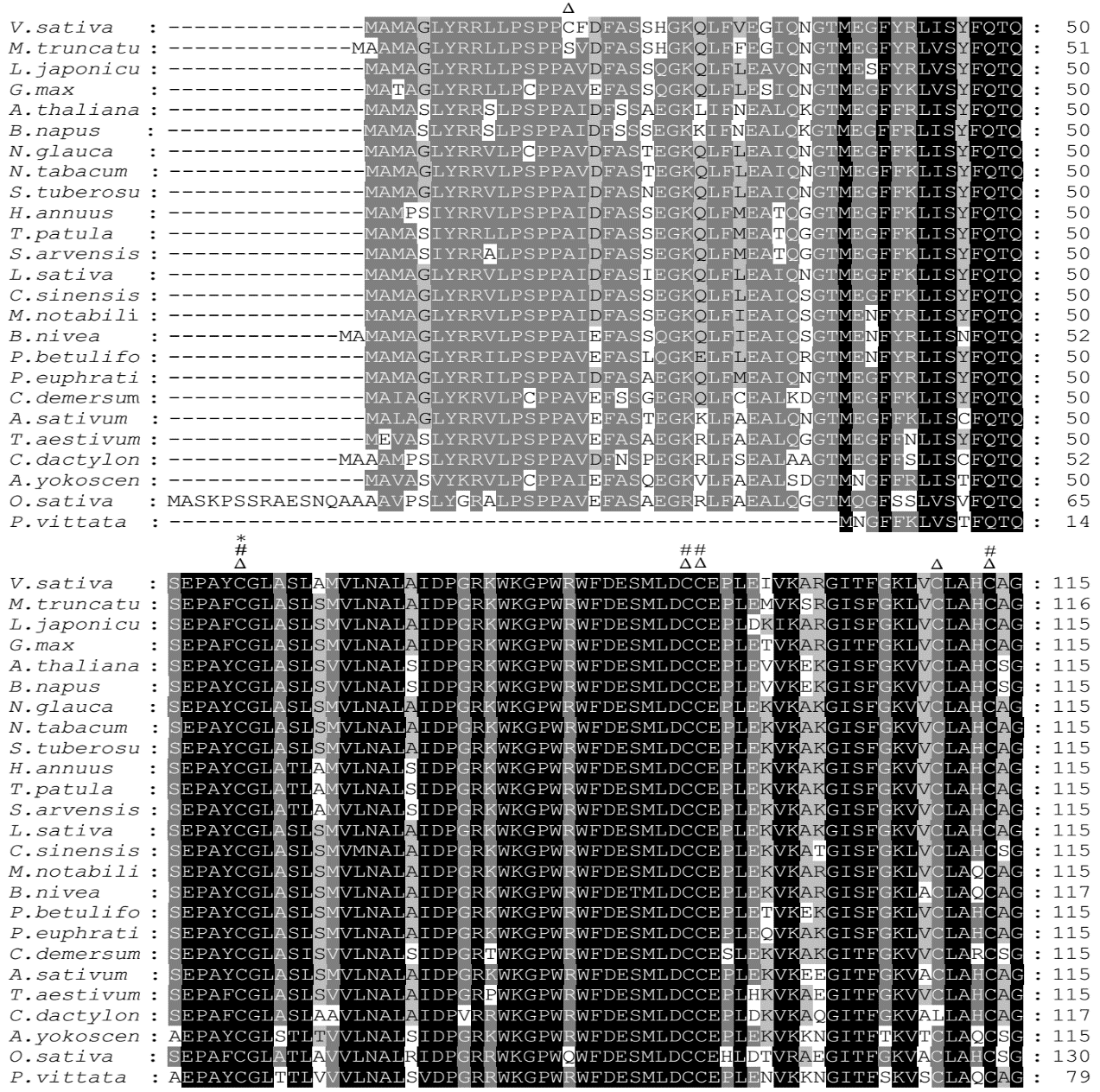

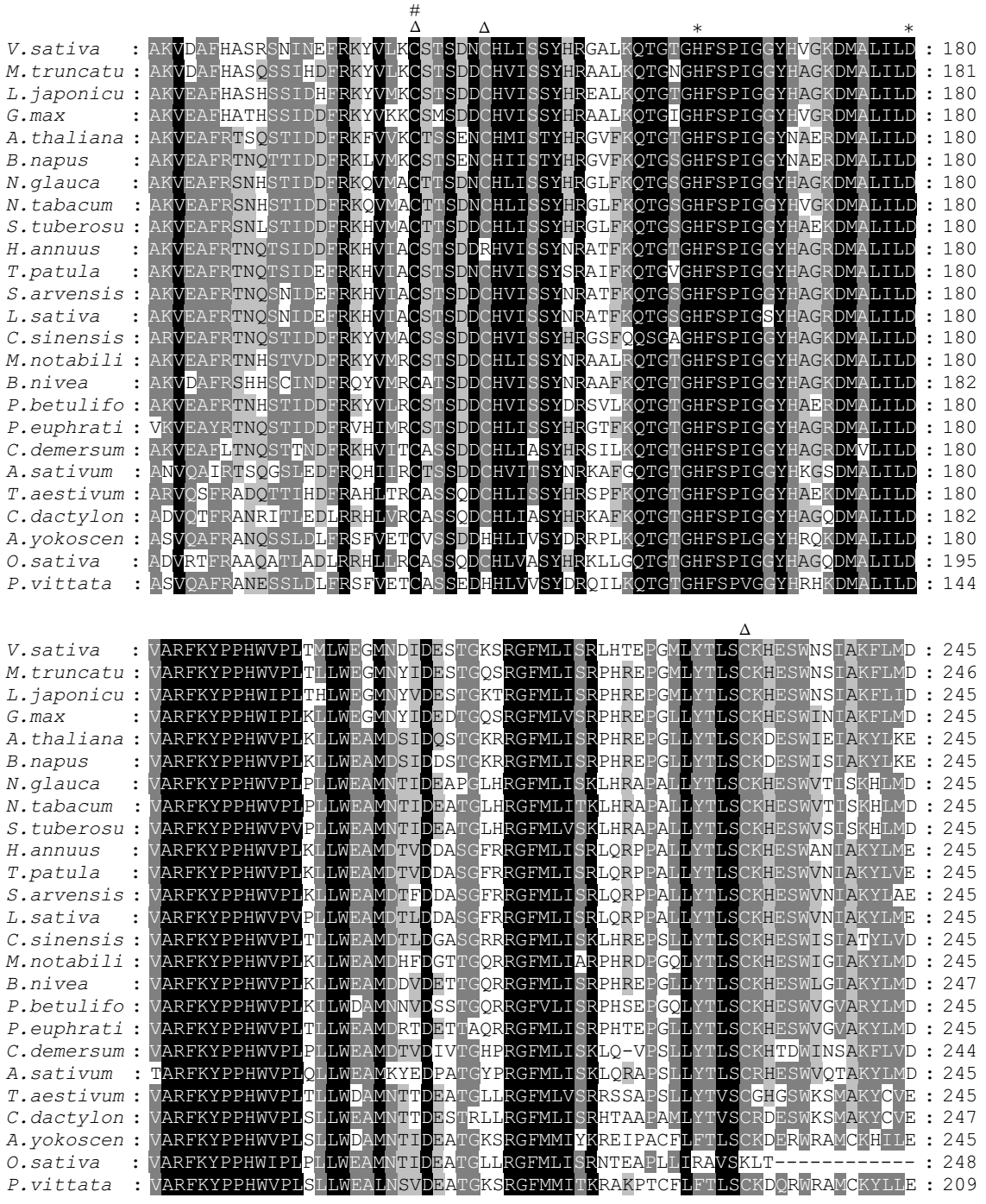

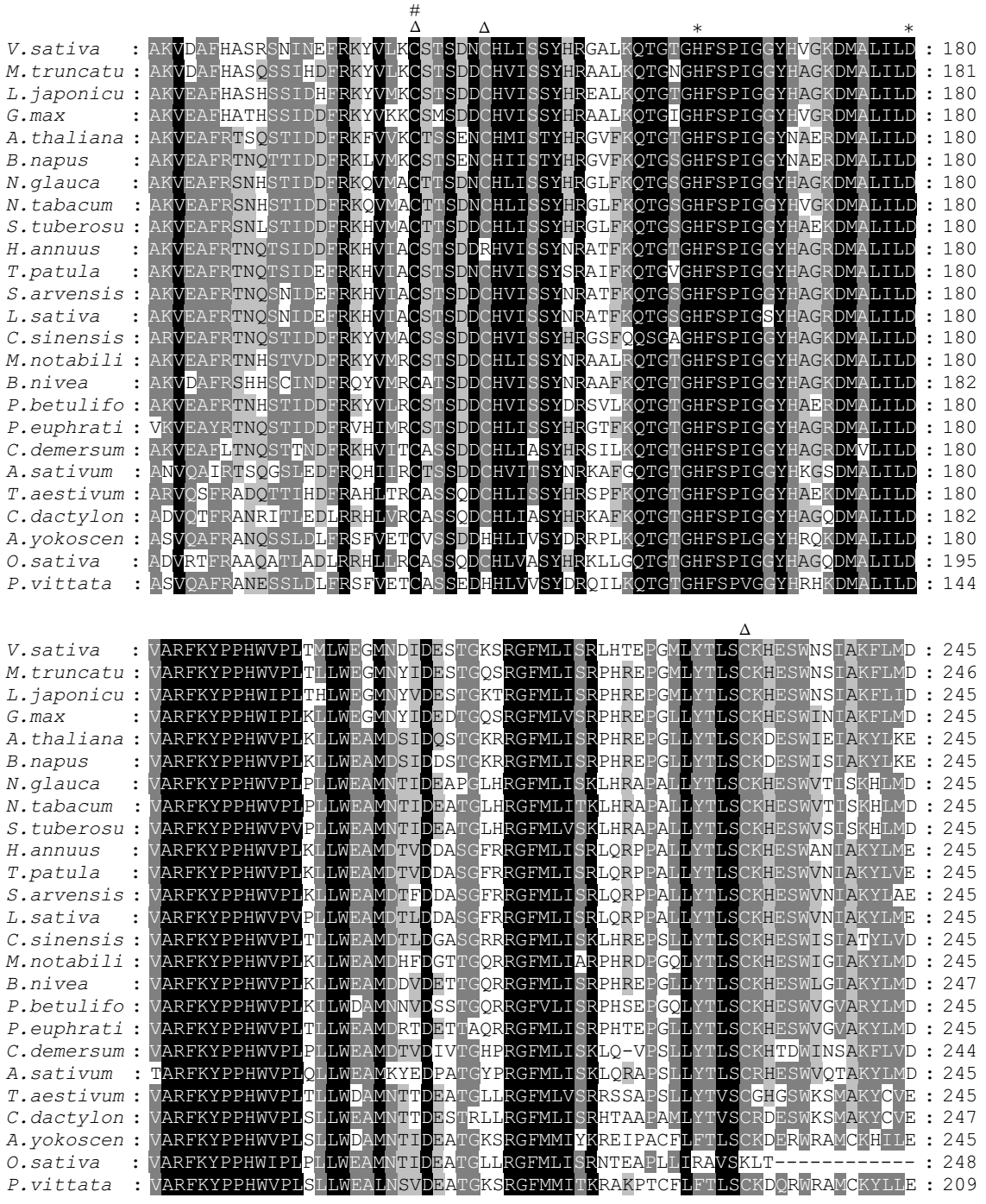

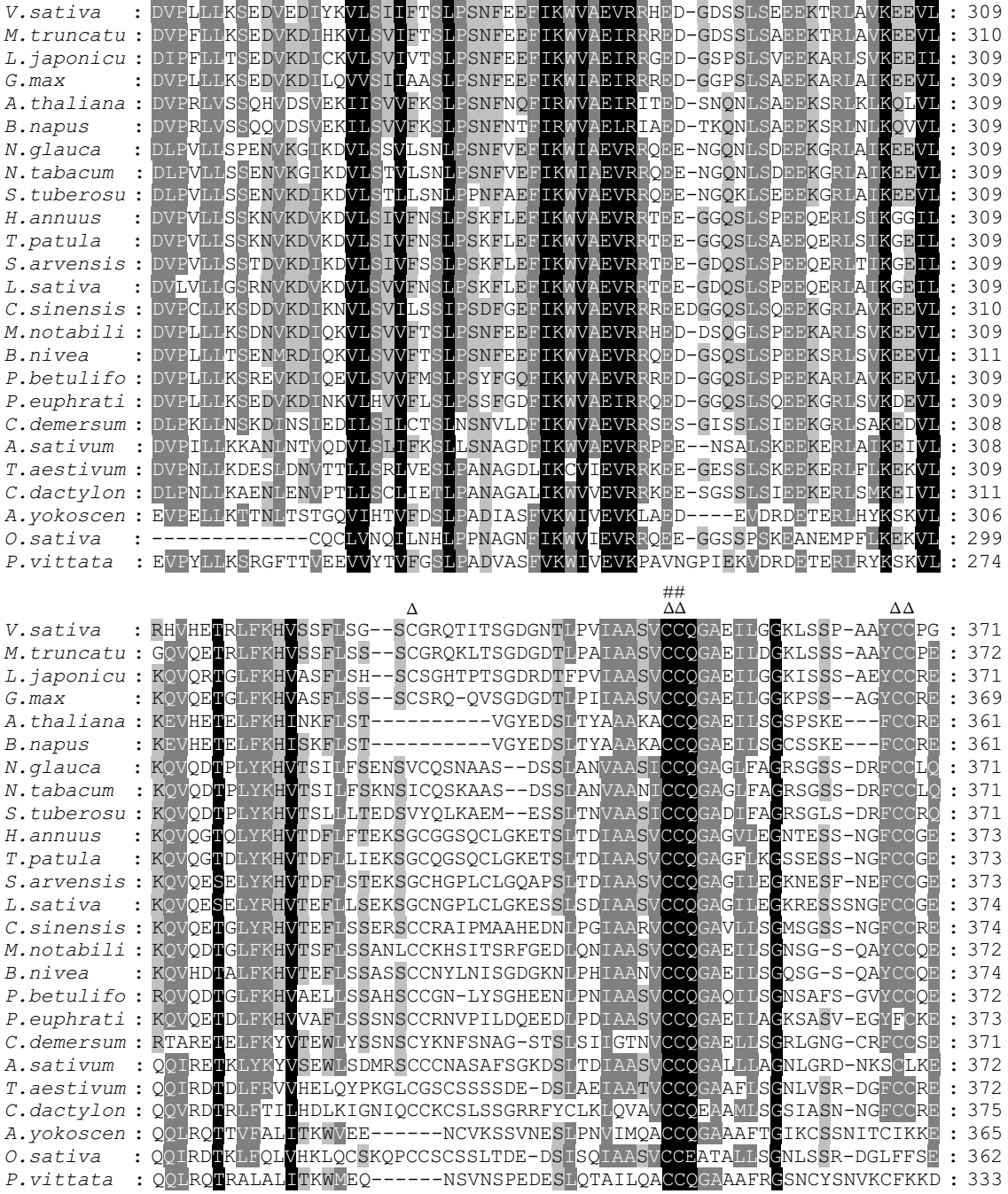


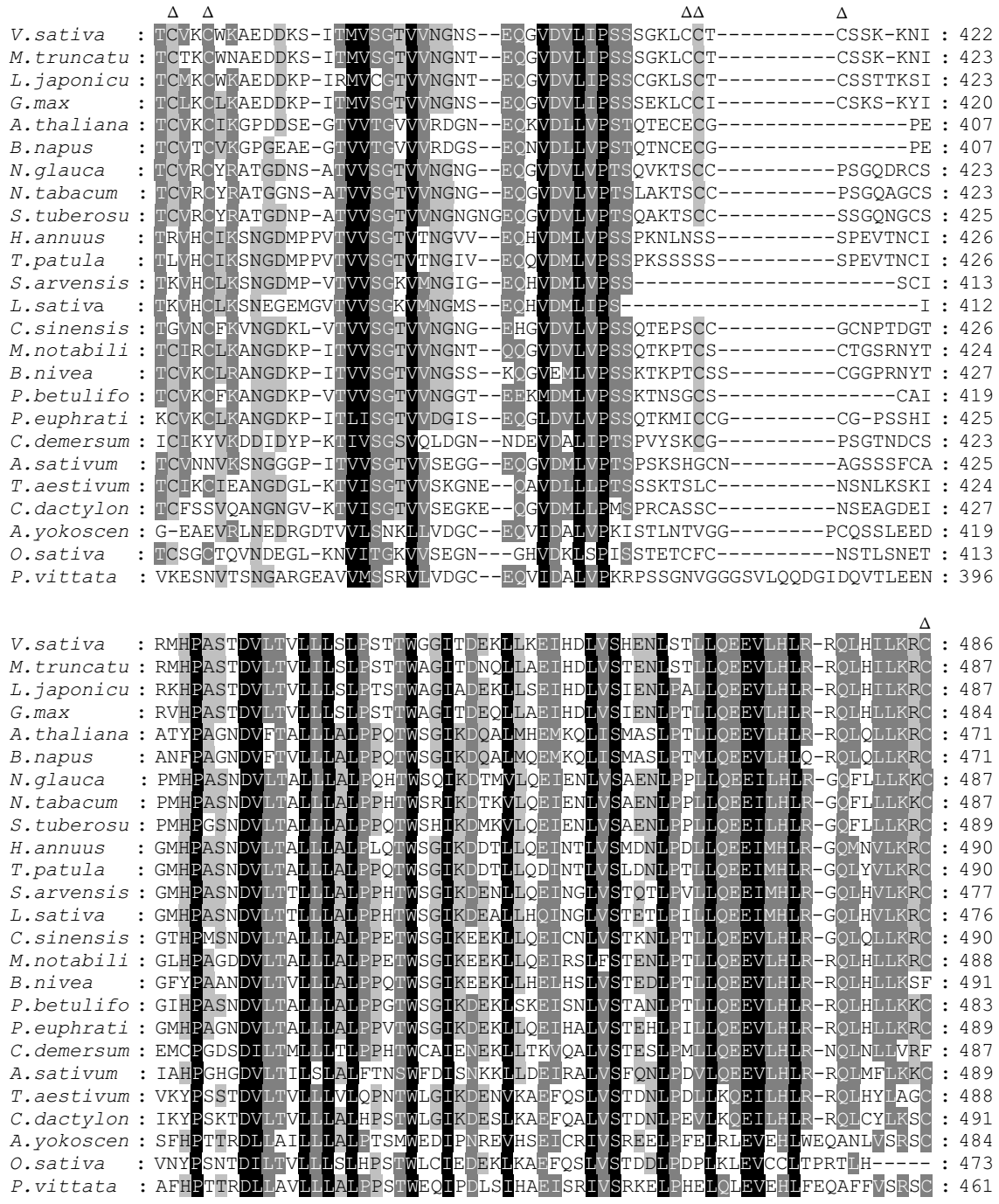


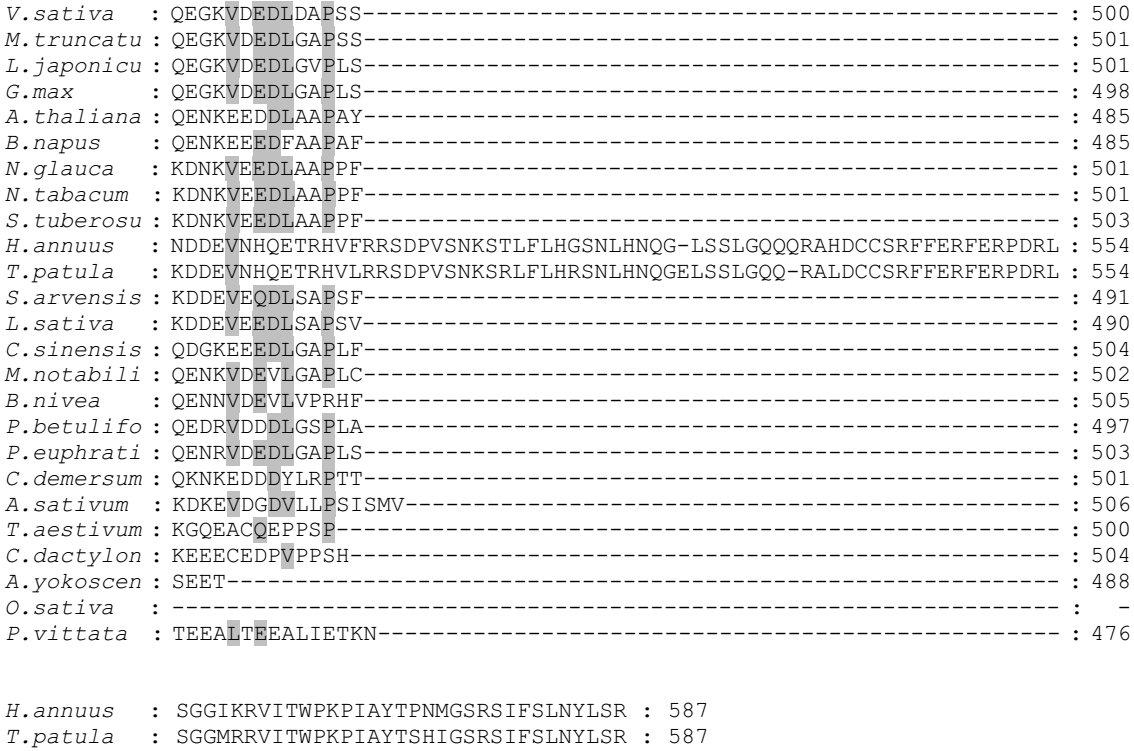


**Supplementary Figure 1.** Sequence analysis of PCS1. Alignment of the amino acid sequences of PCS1 from *V. sativa*, *A. yokoscense* (BAB64932.1), *A. thaliana1* (AAD50593.1), *B. nivea* (AHC98018.1), *B. napus* (AOV94290.1), *C. sinensis* (ARJ31385.1), *G. max* (NP_001235576.1), *H. annuus* (OTG28437.1), *L. japonicus* (AAQ01752.1), *M. notabilis* (AMR70492.1), *M. truncatula* (XP_013449920.1), *N. glauca* (ABX10958.1), *O. sativa* (AAO13349.2), *P. betulifolia* (AEY68569.1), *S. arvensis* (ACU44656.1), *T. aestivum* (AAD50592.1), *L. sativa* (AAU93349.1), *N. tabacum* (AAO74500.1), *C. demersum* (ADR10438.1), *A. sativum* (AAO13809.1), *C. dactylon* (AAO13810.2), *P. vittata* (ADR51707.1), *S. tuberosum* (NP_001275308.1), *T. patula* (AQT18915.1) and *P. euphratica* (XP_011010425.1). Alignment of amino acid sequences was performed using clustalx 1.83. *, Amino acid residues are involved in catalytic active site; #, Cys residues present in plants PCS1 protein; Δ, Cys residues present in VsPCS1 protein.


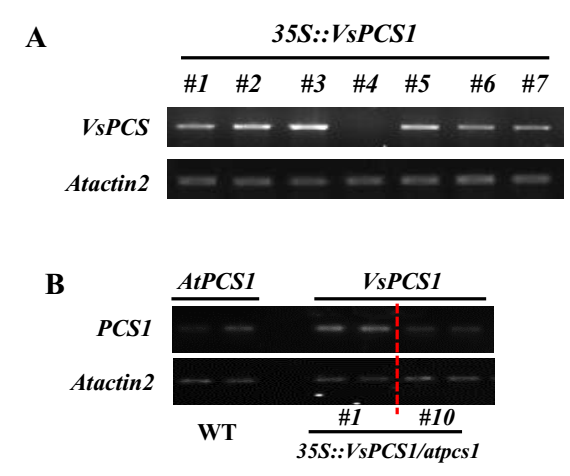


**Supplementary Figure 2.** Expression of *VsPCS1* in transgenic *35S*::*VsPCS1* (A) and *35S*::*VsPCS1/atpcs1* lines (B). Transcripts in 3 week-old seedlings were analyzed using RT-PCR.

**
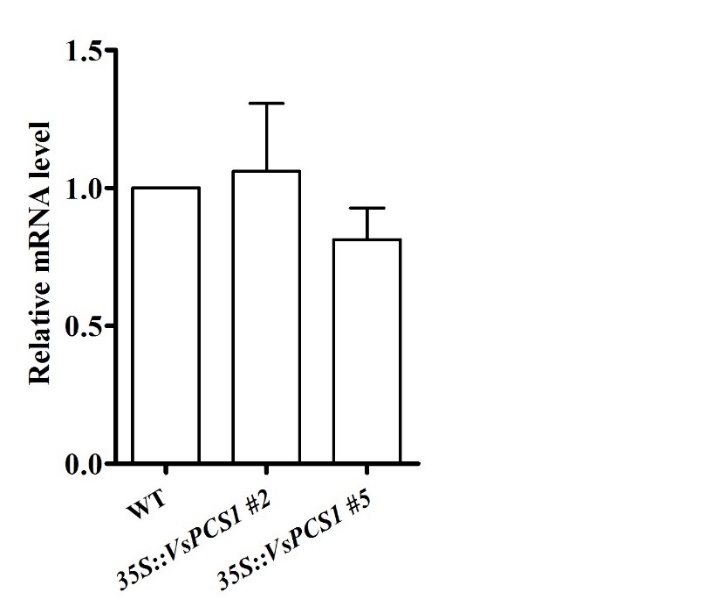
**

**Supplementary Figure 3.** Expression of *AtPCS1* in WT and transgenic *35S*::*VsPCS1* lines. Transcripts in 3 week-old seedlings were analyzed using qRT-PCR.

**
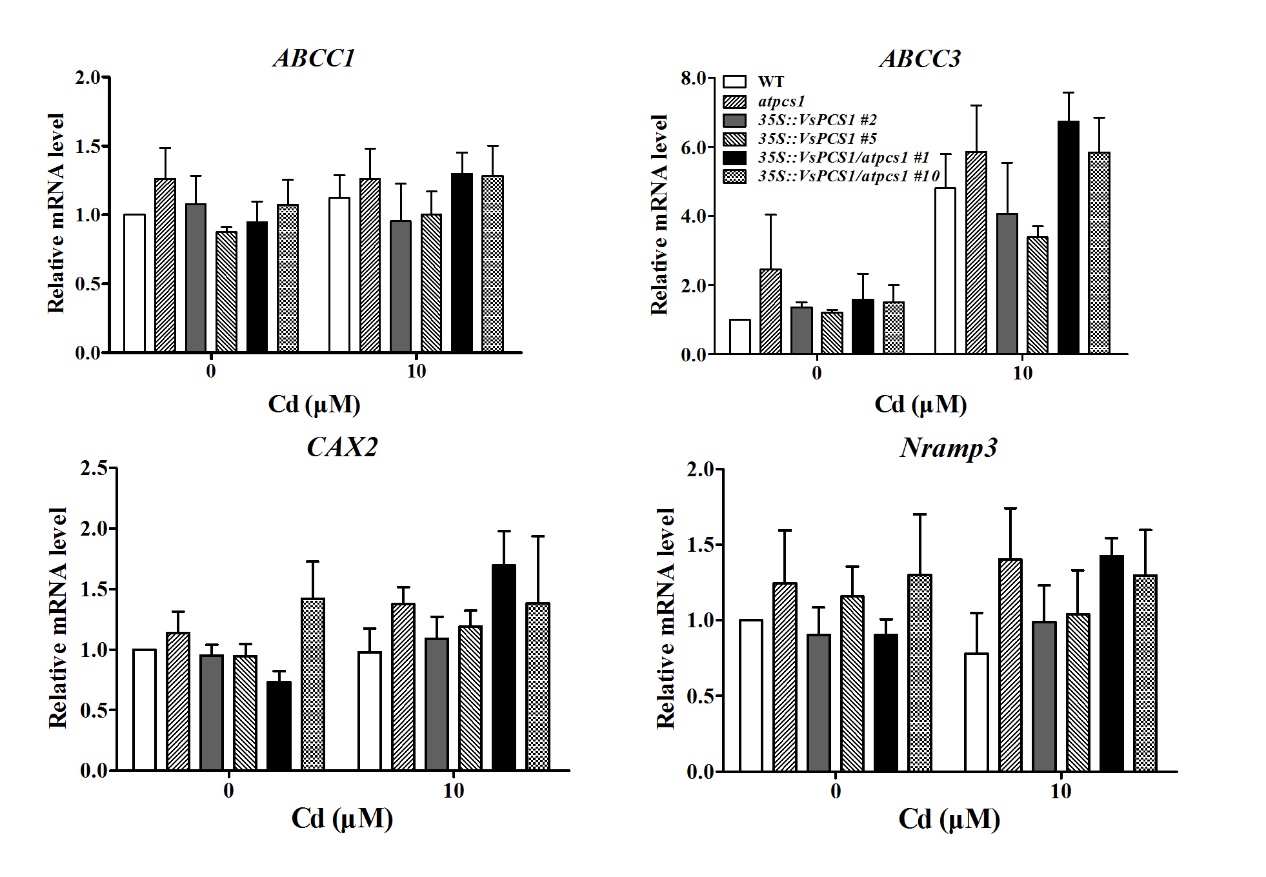
**

**Supplementary Figure 4.** Effects of Cd treatment on gene expression of *AtCAX2, AtNRAMP3* in different Arabidopsis. Seedlings were exposed to 0 and 10 μM CdCl_2_ for 24 h. Total RNA was prepared from the treated tissues was analyzed by quantitative RT-PCR. *Atactin2* was used as an internal control. Values are the means ± standard deviation (SD).

## Supplementary tables

**Supplementary Table 1.** Primers from Arabidopsis used in this study

| Primer name | Sequence 5′ to 3′ |
| --- | --- |
| BP | GCTTCCTATTATATCTTCCCAAATTACCAATACA |
| AtPCS1-R | TTAATCTACCGCAGCAATTGG |
| AtPCS1-F | TGTTTTGAATGTAAGCCTTTGG |
| qAtPCS1-F | GGAAGCCATGGACAGTATTG |
| qAtPCS1-R | TTCTCCTCTGCGCTGAGATT |
| qAtCAX2-F | CTACAAGTTTCCCCCAGCACAC |
| qAtCAX2-R | CAAGAACACCCACCCCTTACTATC |
| qAtNramp3-F | TACTGCTCTTGATTGTTTCGTCTTC |
| qAtNramp3-R | TGAGACTCCCATTGTAGCGATAAG |
| qAtABCC1-F | ACCCTCTTGTTTGTGTATTTCCC |
| qAtABCC1-R | CTCTTCATACTCGTAATCCTCGG |
| qAtABCC3-F | CTTCAGGTCCGATATGCTCCA |
| qAtABCC3-R | TGTTATTCCTCGCAACACAAGAG |
| qAtActin2-F | CTCCTGAAGAGCACCCTG |
| qAtActin2-R | CCCTCGTAGATTGGCACA |

Supplementary Table 2. Primers from *V. sativa* used in this study

| Primer name | Sequence 5′ to 3′ |
| --- | --- |
| PCS1-F1 | ACGCGTCGACATGGCGATGGCGGGGTTGTAT |
| PCS1-R1 | CCGTCATGACTCCTCCTCCAGATGAAGGAGCATCAAGATC |
| PCS1-F2 | GACTAGTATGGCGATGGCGGGGTTGTAT |
| PCS1-R2 | GGACTAGTCTAAGATGAAGGAGCATCAAGATC |
| VsActin11-F | GAGATGAGCGTTTCAGATGTCC |
| VsActin11-R | GTTACCATACAAGTCTTTCCTGAT |
| qVsPCS-F | GGCAGACAGACAATAACTTCAG |
| qVsPCS-R | TCTTCGGCTTTCCAACATTTC |
